# A Reverse Engineering Approach to Optimize Experiments for the Construction of Biological Regulatory Networks

**DOI:** 10.1371/journal.pone.0075931

**Published:** 2013-09-19

**Authors:** Xiaomeng Zhang, Bin Shao, Yangle Wu, Ouyang Qi

**Affiliations:** 1 The State Key Laboratory for Artificial Microstructures and Mesoscopic Physics, School of Physics, Peking University, Beijing, China; 2 The Center for Quantitative Biology and Peking-Tsinghua Center for Life Sciences, Peking University, Beijing, China; Leibniz-Institute for Farm Animal Biology (FBN), Germany

## Abstract

One of the major objectives in systems biology is to understand the relation between the topological structures and the dynamics of biological regulatory networks. In this context, various mathematical tools have been developed to deduct structures of regulatory networks from microarray expression data. In general, from a single data set, one cannot deduct the whole network structure; additional expression data are usually needed. Thus how to design a microarray expression experiment in order to get the most information is a practical problem in systems biology. Here we propose three methods, namely, maximum distance method, trajectory entropy method, and sampling method, to derive the optimal initial conditions for experiments. The performance of these methods is tested and evaluated in three well-known regulatory networks (budding yeast cell cycle, fission yeast cell cycle, and E. coli. SOS network). Based on the evaluation, we propose an efficient strategy for the design of microarray expression experiments.

## Introduction

One of the main fields in biological researches is to reveal biological regulatory networks that control different functions. In the past, molecular interactions have been established at a rather slow pace. For example, it took more than a decade from the discovery of the well-known tumor suppressor gene p53 to the establishment of its regulatory feedback loop with the protein MDM2 [1]. This situation has been qualitatively changed thanks to the development of different new biotechnologies, especially microarray expression experiments. Accordingly, theoretical systems biology has provided several algorithms for the deduction of regulatory interactions from experimental data. These algorithms can be employed to effectively reconstruct biological regulatory networks. One of the successful network reconstruction methods is reverse engineering approach [2], [3], [4], [5], [6], [7], [8], [9], [10], which has been advancing very rapidly in recent years [11]. At present, there are mainly four types of the reverse engineering algorithms: correlation-based methods [7], information-theoretic methods [9], Bayesian network predictions [6], and methods based on dynamic models [4], [5]. In this paper, we apply the simplest method, the correlation-based Boolean reverse engineering, to discuss algorithms for the optimization of experiments in network construction.

The basic procedures of the Boolean reverse engineering method are given as follows: Firstly, starting from experimental data (i.e. the mRNA expression level as a function of time), one reduces the analog experimental data into a digital (0 or 1) Boolean type of time sequence, which can be represented as a trajectory in phase space. Secondly, one defines the dynamic rules of network interactions. In the case of Boolean dynamics, the interaction between node *i* and node *j* can be simplified into an interaction coefficient (*a_ij_*), and three types of interactions can be defined: inhibition (*a_ij_*<0), activation (*a_ij_>0*), and no interaction (*a_ij_ = 0*). The evolution of the system’s state can be described by Boolean dynamics equations, such as the following:
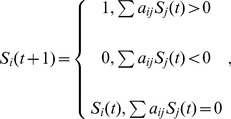
(1)


where *S_i_(t)* represents the state of node *i* at time *t*; {*a_ij_*} forms the to-be –determined regulatory matrix of the network. Finally, one uses logical expressions (see Method) to construct the regulatory matrix under the restriction of the trajectory obtained from the mRNA expression data.

In general, the information from one experiment (one trajectory) is insufficient for the reconstruction of the underlying control network; many possible networks can generate the same trajectory under the same dynamic rules of Eqn. (1). So that additional experiments (more trajectories) are needed to identify the real regulation network (in the case of Boolean dynamic, the regulation matrix). Every additional experiment will generate new information on the control network, so that it helps to cut-down the number of the possible networks. Different trajectories, however, result in different efficiencies. The purpose of this study is to develop algorithms to design experiments so that one can use minimal number of experiments to identify the underlying regulation network. Instead of using data from experiments, in this study we applied three well-known regulatory networks (budding yeast cell cycle, fission yeast cell cycle, and E. coli. SOS network) as tests to generate the trajectories of the networks according to Eqn. (1). The reconstructed networks were compared with the original ones to evaluate the algorithms. One of the possible ways to obtain different trajectories is to change the initial conditions of the dynamic system. As a demonstration, we used this way to obtain different trajectories in this study.

## Materials and Methods

### Boolean model

**Figure 1:**
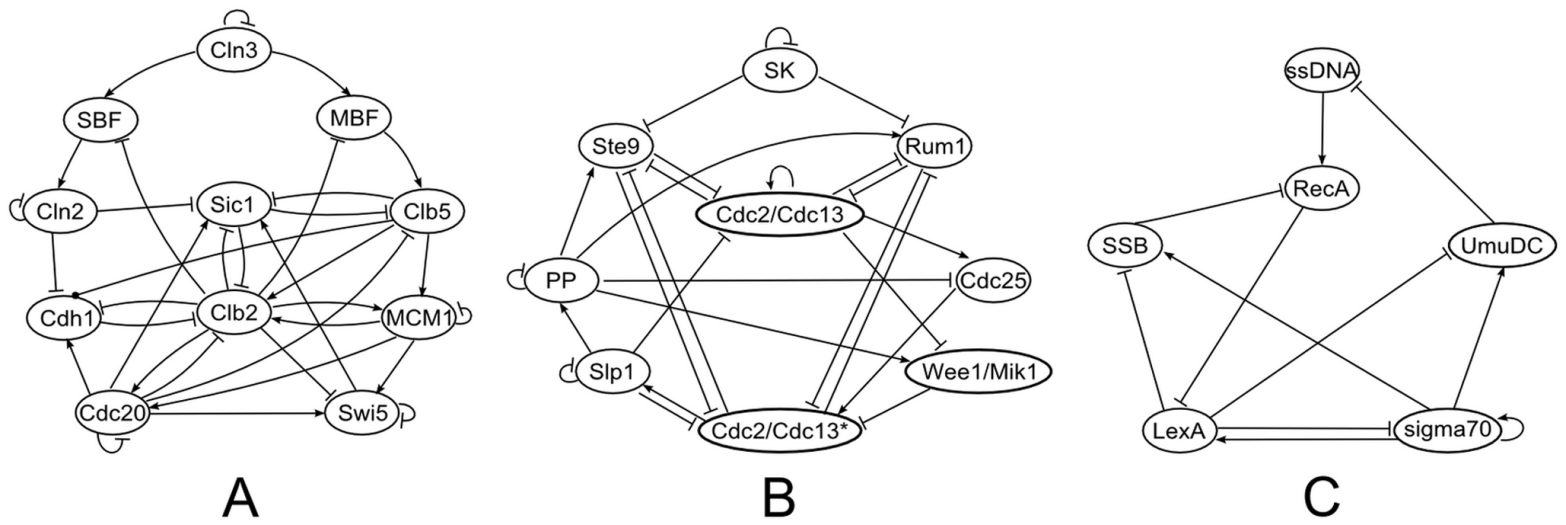
The regulatory networks. A, the network of mitotic cell cycle of budding yeast; B, the network of mitotic cell cycle of fission yeast; C, The network of SOS network of *E.coli*. The nodes represent the essential proteins. The lines with bar ending represent repression. The lines with arrow ending represent activation. One node only has two states: 0 (inactive) and 1(active).

For regulatory networks as shown in Fig. 1, their dynamics can be described with different modeling approaches. The most frequently studied models include stochastic model (master equation), continuous model (ordinary/partial differential equation), and discrete model (difference equation). Once initial (boundary) conditions are given, these equations provide a unique trajectory in phase space. Among different models, the most straightforward one is the Boolean model. Previous study found that this model can catch the essence of the dynamics of the control networks [12]. As stated previously, the Boolean model simplifies a biological species (DNA, mRNA or protein) to a node; the state of each node can be 0 or 1, 0 being inactive and 1 being active. The interactions of the network are represented by links, and the types of interactions are reflected by the interaction coefficient: *a_ij_* = −∞ for inhibition (for dominant inhibition model), *a_ij_* = 0 for non-interaction, and *a_ij_* = 1 for activation. The interactions form a regulatory matrix. Given an initial condition, the sequence of states at different times can be calculated using Eqn. (1), which we call a trajectory. Table 1, Table 2 and Table 3 provide the normal trajectories (calculated using natural initial conditions) of budding yeast cell cycle (Table 1), fission yeast cell cycle (Table 2), and *E. coli* SOS network (Table 3). They were calculated using the regulatory networks presented in Fig. 1A, 1B, and 1C respectively. It should be noted that for the inhibition interaction, the value of *a_ij_* was set to −∞ here instead of −1 in previous work [12], in order to emphasize the fact that the effects of inhibitors are always stronger than that of activators from a biological point of view.

**Table 1:** The biological pathway of budding yeast cell cycle.

	Cln3	SBF	MBF	Cln2	Cdh1	Swi5	Cdc20	Clb5	Sic1	Clb2	Mcm1
1	1	0	0	0	1	0	0	0	1	0	0
2	0	1	1	0	1	0	0	0	1	0	0
3	0	1	1	1	1	0	0	0	1	0	0
4	0	1	1	1	0	0	0	0	0	0	0
5	0	1	1	1	0	0	0	1	0	0	0
6	0	1	1	1	0	0	0	1	0	1	1
7	0	0	0	1	0	0	1	1	0	1	1
8	0	0	0	0	0	0	1	0	0	0	1
9	0	0	0	0	1	1	1	0	1	0	0
10	0	0	0	0	1	1	0	0	1	0	0
11	0	0	0	0	1	0	0	0	1	0	0

**Table 2:** The biological pathway of fission yeast cell cycle.

	SK	Cdc2	Ste9	Rum1	Slp1	Cdc2*	Wee1	Cdc25	PP
1	1	0	1	1	0	0	1	0	0
2	0	0	0	0	0	0	1	0	0
3	0	1	0	0	0	0	1	0	0
4	0	1	0	0	0	0	0	1	0
5	0	1	0	0	0	1	0	1	0
6	0	1	0	0	1	1	0	1	0
7	0	0	0	0	1	0	0	1	1
8	0	0	1	1	0	0	1	0	1
9	0	0	1	1	0	0	1	0	0

**Table 3:** The biological pathway of *E.coli* SOS network.

	ssDNA	RecA	LexA	Sigma70	UmuDC	SSB
1	1	0	1	0	0	0
2	1	1	1	0	0	0
3	1	1	0	0	0	0
4	1	1	0	1	0	0
5	1	1	0	1	1	1
6	0	0	0	1	1	1
7	0	0	1	1	1	1
8	0	0	1	0	0	0

### Reverse engineering of Boolean model

In the reverse engineering of Boolean model, we ask a reverse question: Given an evolution trajectory of a regulatory system such as shown in Table 1, Table 2 and Table 3, what is the underlying regulation network that can perform this function? More specifically, we try to derive the control matrix of the Boolean model based on the sequence of states at different times. Here, we use the mathematical formula of Ref.[13] to address the question. In this formula, the network interactions are treated as logical variables. If there is an inhibition interaction from node *i* to node *j*, we note *r_ij_* = TRUE, otherwise *r_ij_* = FALSE. Similarly, if there is activation interaction from node *i* to node *j*, we note *g_ij_* = TRUE, otherwise *g_ij_* = FALSE. With a Boolean model incorporating strong inhibition (*a_ij_* = −∞), one can translate the trajectory constraint into a logical expressions [13].



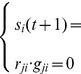
(2)


where the ‘+’ and ‘∑’ denote the logic function ‘OR’; the ‘

’ and ‘∏’ denote logic function ‘AND’; the bar denotes ‘NOT’; 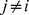 ranges from 1 to N, N being the number of nodes of the network. States of all nodes in the trajectory should satisfy Eqn. (2) at all times. Therefore, the ensemble of Eq. (2) for different nodes and at different evolution time, provides the logical expression of the trajectory constraint. After some mathematical transformation and simplification, one can obtain the Conjunctive Normal Form (CNF) or k-set form of the expression, which is the multiplication of summation of items. The CNF can be easily analyzed to obtain meaningful information of the regulatory network[13]. For example, in the trajectory of Table 3, according to Eq. (2), the logic constraint of 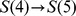 for the node Sigma70 can be expressed as



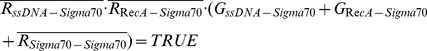
.

In this expression, separated items indicate the presence of an edge, separated items with a bar indicate the absence of an edge, and items in parentheses indicate the presence of at least one combination of these. Thus the above expression implies that there must not be an inhibition link from node *ssDNA* to node *Sigma70*; there must not be an inhibition link from node *RecA* to node *Sigma70*; and there is at least one activation link to node *Sigma70*, either from node *ssDNA* or node *RecA*, or no self**-**inhibition link of *Sigma70*.

**Figure 2:**
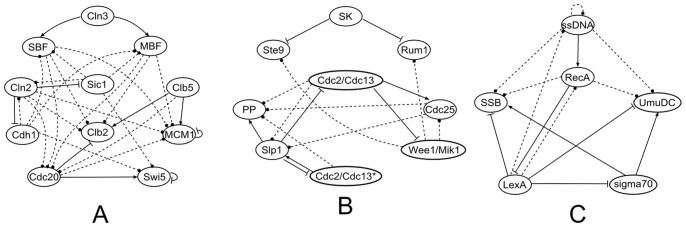
The results of reverse engineering analysis. The interactions must (solid line) and must not (dashed line) exist in the networks. A, for budding yeast cell cycle network; B, for fission yeast cell cycle network. C, for *E.coli* SOS network.

Putting the constrain of all the nodes at all steps of Table 3 together, we obtain the logical expression as the following



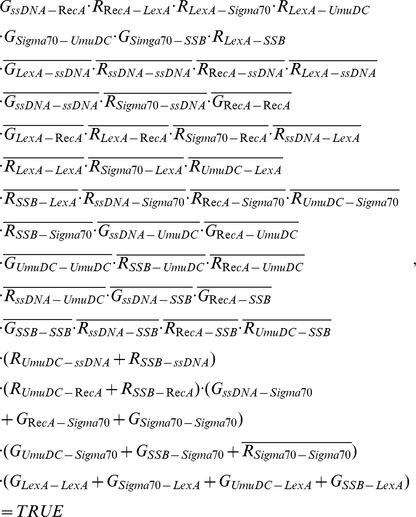



which contains all the information derivable from the trajectory constraint. By interpreting this formula in terms of network components, the must-exist edges and must-not-exist edges can be identified, as shown in Fig. 2C. In total, 7 out of 10 existing interactions have been established from SOS response trajectory. However the undetermined edges all together correspond to about 7.1×10^6^ possible networks. The same procedure can be applied to the trajectories of budding yeast cell cycle (Table 1) and fission yeast cell cycle (Table 2), where the must-exist edges and must-not-exist edges are shown in Fig. 2A and Fig. 2B respectively. The undetermined edges all together give out about 2.8×10^31^ possible networks for budding yeast cell cycle and about 9.6×10^21^ possible networks for fission yeast cell cycle.

### Applying Reverse Engineering Method to *in silico* data

We also test this method on *in silico* data set from DREAM4 challenge[14] and find out that our method can regenerate major regulations of the network. Our work mainly focuses on the first network with 10 genes in Dream 4 challenge. The connection matrix of the network is presented in Table 4. We use the wild type steady state of all the genes as baseline (Table 5), and combine knock-out data (Table 6) and time-series data to test our method.

**Table 4:** Connection matrix of the first gold standard network in Dream 4 challenge.

	G1	G2	G3	G4	G5	G6	G7	G8	G9	G10
G1	0	−1	1	1	−1	0	0	0	0	0
G2	0	0	0	0	0	0	0	0	0	0
G3	0	0	0	1	0	0	−1	0	0	0
G4	0	0	−1	0	0	0	0	0	0	0
G5	0	0	0	0	0	0	0	0	0	0
G6	0	−1	0	0	0	0	0	0	0	0
G7	0	0	1	1	0	0	0	0	0	0
G8	0	−1	0	0	0	−1	0	0	0	0
G9	0	0	0	0	0	0	0	0	0	1
G10	0	0	−1	1	0	0	0	0	0	0

**Table 5:** Wild type steady state in Dream4 data set.

G1	G2	G3	G4	G5	G6	G7	G8	G9	G10
0.60	0.12	0.33	0.60	0.15	0.33	0.50	0.65	0.61	0.75

**Table 6:** Knock out data set, all the diagonal elements are 0.

	G1	G2	G3	G4	G5	G6	G7	G8	G9	G10
G1 knock out	0.00	0.37	0.26	0.36	0.80	0.38	0.83	0.77	0.62	0.72
G2 knock out	0.73	0.00	0.38	0.60	0.15	0.34	0.52	0.68	0.74	0.79
…	0.71	0.14	0.00	0.37	0.08	0.40	0.66	0.80	0.72	0.67
…	0.85	0.11	0.62	0.00	0.15	0.28	0.11	0.63	0.59	0.72
…	0.88	0.14	0.43	0.56	0.00	0.33	0.54	0.57	0.73	0.79
…	0.62	0.40	0.46	0.63	0.09	0.00	0.46	0.59	0.74	0.80
…	0.69	0.09	0.32	0.27	0.08	0.30	0.00	0.75	0.80	0.64
…	0.80	0.28	0.38	0.66	0.12	0.63	0.55	0.00	0.77	0.69
…	0.79	0.12	0.24	0.40	0.14	0.30	0.69	0.77	0.00	0.04
…	0.79	0.14	0.42	0.36	0.12	0.34	0.62	0.68	0.72	0.00

#### Discretization of data

We first use the following rules to discretize the data: If expression of Gene X in some knock out data set is 0.2 larger (smaller) than the baseline, then we set the value of Gene X in those data set to 1(0), the baseline value and the rest of knock out data is set to 0(1) accordingly. If there’s no significant difference between baseline value and expressions in knock out strains. Then all the values are set to be 1. We suspect baseline value of Gene 1 is too low, so all values of Gene 1(except in G 1 knock out) is set to 1. Taking Gene 9 and Gene 10 for example, all values in 9^th^ column (except Gene 9 knock out) is in the +/− 0.2 range of baseline level, then all the expressions of Gene 9 are set to 1. For Gene 10, the baseline level is set to 1, and the value of Gene 10 in Gene 9 knock out strain is set to 0. This restriction may be relaxed in later improvement of our method. After discretization, we noticed that the steady state (Table 7) is the largest attractor of the network of Table 4 when we use Boolean network model to simulate the network. The result of analysis is shown in Table 8.

**Table 7:** The wild type steady state after discretization.

G1	G2	G3	G4	G5	G6	G7	G8	G9	G10
1	0	0	1	0	0	0	1	1	1

**Table 8:** Four largest attractors of the network.

size	G1	G2	G3	G4	G5	G6	G7	G8	G9	G10
WT	1	0	0	1	0	0	0	1	1	1
104	1	0	0	1	0	0	0	1	1	1
56	1	0	0	1	0	0	0	1	0	0
52	1	0	0	1	0	0	0	0	1	1
52	0	0	0	1	0	0	0	1	1	1

When we learn the regulators of Gene X from knock out data, we use the 9 different steady states (except Gene X knock out) to deduct the network structure. A modification of discretization is used: level of Gene X is determined according to the former procedure, but for the other genes, its knock out value is set to 0, all expressions larger than 0.2 are set to 1.(when Gene Y is knock out, its expression level is often significantly lower than its baseline level). In this way, we get the discretized knock-out data (Table 9).

**Table 9:** Steady States used to get regulators of Gene 10.

	G1	G2	G3	G4	G5	G6	G7	G8	G9	G10
G1 knock out	0	1	1	1	1	1	1	1	1	1
G2 knock out	1	0	1	1	0	1	1	1	1	1
…	1	0	0	1	0	1	1	1	1	1
…	1	0	1	0	0	1	0	1	1	1
…	1	0	1	1	0	1	1	1	1	1
…	1	1	1	1	0	0	1	1	1	1
…	1	0	1	1	0	1	0	1	1	1
…	1	1	1	1	0	1	1	0	1	1
G9 knock out	1	0	1	1	0	1	1	1	0	0

#### Time series data

Time series data is discretized according to its value in comparison with baseline (wild type value). Adjacent states are compressed if they are same. It turns out that time series data provides little information (Tables 10 and 11). There’re 5 trajectories in total. But we only use time series 1, 3, and 4, because the rest is either too noisy or lack useful information.

**Table 10:** First time trajectory after discretization (t = 500 to t = 1000).

time	G1	G2	G3	G4	G5	G6	G7	G8	G9	G10
1	1	0	0	1	1	0	0	1	1	1
2	1	0	0	1	0	0	0	1	1	1

**Table 11:** Third time trajectory after discretization (t = 500 to t = 1000).

time	G1	G2	G3	G4	G5	G6	G7	G8	G9	G10
1	1	0	0	1	1	1	0	1	1	1
2	1	0	0	1	0	0	0	1	1	1

#### Inference of Network

Using knock out data alone, we can get an ensemble of all possible networks. The total number depends on the specific knock-out data, but it is all around 10^19^, comparable with the number of possible networks of fission yeast cell cycle. After randomly sampling 10000 networks (we choose this number because the possibilities of edges have converged) from this ensemble and calculating the possibilities of each edge. The method can deduct 6 most possible links. Compared with the right network of Table 4, we find out that among the 6 most possible edges, four is correct. They are 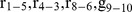 (the wrong ones are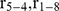). After we use the time series data in addition, possibility of 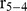 goes to zero. Thus we get a Positive Predictive Value (PPV) of 4/5 = 0.8. However, there still exists a large number of possible networks (about 10^19^). The question now is how to select another trajectory (using another initial condition) so that the number of possible networks can be minimized.

### Reduction methods

To reduce the number of possible networks, we need extra information from other experiments (trajectories), which in principle can be provided by choosing different initial conditions. In this work, instead of using data from experiments, we used the actual networks shown in Fig. 1 and the dynamic rule of Eq. (1) to generate new trajectories. Three methods in choosing initial condition were considered. The performance of each method was evaluated for each network.

#### Maximum distance method

The basic idea of this method is that the new trajectory that generated from newly selected initial condition should have the least overlap with the trajectories that we have already considered. Greater difference between this trajectory and the other trajectories implies a larger amount of information that extractable from the new trajectory. For this purpose, we used the following strategy to select initial condition in experiment. First we defined the distance between two molecular states:



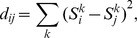
where 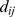 denotes the distance between state *i* and state *j*, and 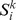 denotes the state of protein *k* in network state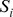. Furthermore, we defined the shortest distance of an initial state 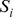
to the known trajectory 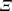:



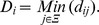



We speculated that a longer distance of an initial condition to the known trajectories will generate a new trajectory that contains greater amount extractable information, so that we select the state with the longest distance D to the known trajectories as the initial condition:
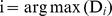



#### Trajectory entropy method

The basic idea of this method is that the new trajectory developed from newly selected initial condition should maximally reduce the uncertainties in network selection. Here, we take the control network of SOS system (Fig. 1C) as an example. The biological trajectory of Table 3 reduces the number of possible networks to 7.1×10^6^. Selecting a new initial condition and applying it to all of these possible networks will produce 7.1×10^6^ trajectories. Some of these trajectories might be identical. We presume that there are k different trajectories, and the trajectory 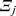
can be produced by *N_j_* networks. We can thus define a value 
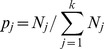
for this trajectory, and the trajectory entropy 
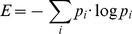
for this initial condition. A larger trajectory entropy corresponds to greater amount of information obtainable from the selected initial condition. Therefore, in principle, one should select the initial condition which yields the largest trajectory entropy. However, in practice, the total number of possible networks is usually too large for computation; the precise entropy can only be obtained when the network number is below 10^7^. In dealing with more complex control networks as shown in Fig. 1A and 1B, one needs to find an alternative to estimate the trajectory entropy.

Our alternative to evaluate the trajectory entropy is to compute it step by step. We let 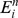 denote the trajectory entropy with initial network state *i* and trajectory length *n*. The first step 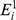
can be easily evaluated. For this purpose, we first add a new constraint: the trajectory goes from state i to state j, then we apply the reverse engineering method (described in the section Reverse engineering of Boolean model) to obtain the remaining network number *n_ij_*. Subsequently we generate a state transfer matrix
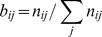
, where *b_ij_* denotes the probability of a transfer from an initial state *i* to a state *j*. It can be proven that the expression of the first step trajectory entropy is given by 
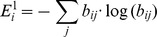
. If we suppose the transfer matrix { *b_ij_* } remains almost the same as states of the system evolves to the next time step, we can obtain the entropy 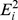:



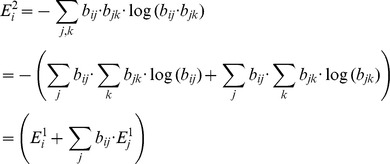
.

With the same assumption, we can obtain 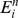 recursively:
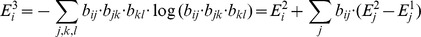




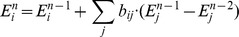
 (n>3).

The computation goes to a stop when 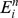 no longer increases with *n*. The final value is considered as the trajectory entropy of initial condition *i*. In this study, we used this alternative way to evaluate trajectory entropy.

#### Sampling method

The above-mentioned alternative way to calculate the trajectory entropy is based on the assumption that the transfer matrix { *b_ij_* } remains unchanged when states evolve. To our knowledge, this assumption has not been proven. The sampling method by-passes this alternative by obtaining a statistic value of trajectory entropy instead. In this method, we chose only a part of networks to obtain the value of *p_j_*, subsequently calculate the trajectory entropy using 
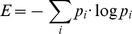
. In the calculation, we doubled the sampling number until the entropy value converges. For SOS network (Fig. 1C), the value of trajectory entropy converges when the sampling number is about 1000; for the budding yeast cell cycle and fission yeast cell cycle networks (Fig. 1A and 1B), the value converges when the sampling number is about 10000.

## Results

**Figure 3:**
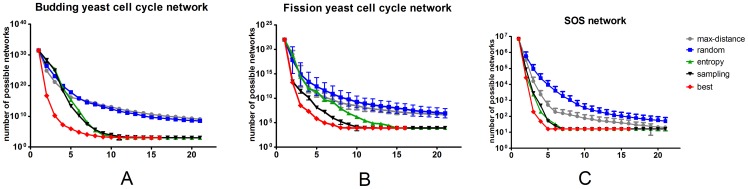
The performances of the three methods compared with random method and the best sequence. The best sequence is calculated by enumerating all the initial conditions. A, in budding yeast cell cycle network; B, in fission yeast cell cycle network; C, in *E.coli* SOS network.

The performance of the three methods in the SOS network of *E. coli*, cell cycle network of budding yeast, and cell cycle network of fission yeast are summarized in Fig. 3. Each line in Fig. 3 shows the remaining number of undetermined networks as a function of number of “experiments” (in this study the number of initial conditions to calculate new trajectories). One observes that the number of undetermined networks decreases as more trajectory information is provided. However, the slope of the curves varies with different methods in determining initial conditions of experiments.

The number of possible networks of *E.coli* SOS network under the constraint of Table 3 is only about 7×10^6^. In this simple case all the three methods can effectively work. The entropy and sampling methods in average decrease the number of remaining network 10 times per step; both of them used about 5 steps to reach the minimum network number. Because of the symmetry of node UmuDC and node SSB, the minimum network number cannot be decreased to one.

As the number of node in a network increases, the differences in performance of different methods become large. This is reflected by Fig. 3A and Fig. 3B. Taking the budding yeast cell cycle network for example, in average each new “experiment” can reduce the number of possible networks by three orders of magnitude for sampling and entropy method, and two orders of magnitude for maximum distance and random method. Maximum distance method has almost the same performance as random method in these two networks, although it performs slightly better in SOS network. The performance of the 3 methods in the same network can be ranked as P_sampling_ ≥P_entropy_ > P_distance_ ≥P_random_.

The calculation cost for the three methods all increases with the increase of the node number of the network; it is ranked as T_entropy_ > T_sampling_ > T_distance_ >T_random_. The calculation cost of entropy method increases very rapidly as the node number increase; it becomes very time-consuming when the node number is large. In contrast, the calculation time of max-distance and sampling methods increase very slowly with the increase of the node number of network. At the same time the performance of sampling method kept satisfyingly, but the performance of entropy and max-distance methods worsened. As a result of these analyses, we conclude that sampling method has the best performance, acceptable level of calculation cost and unique resolution. It is the best among the three methods.

**Figure 4:**
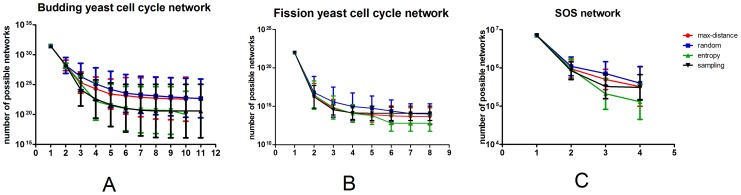
The performances of the three methods when the number of perturbed nodes are limited. For all methods we randomly choose 20 different combinations of perturbed nodes and get the average performance. A, in budding yeast cell cycle network; B, in fission yeast cell cycle network; C, in *E.coli* SOS network.

In practice, not all the initial conditions can be implemented in experiments. To make our algorithms more practicable in a real experiment, we consider a constraint on the number of node that can be perturbed. For each network, we assume only 35% of all the nodes can be modulated. For example, we can perturb 3 nodes in fission cell cycle network, making the total number of possible initial condition equals to 2^3^ = 8. In order to reflect the effects of different combinations of the perturbed nodes, we run each algorithm for 20 times and get the average performance (Figure 4). In budding yeast network, sampling method outperforms the other methods, though the error bar is large (partly due to the various combinations of perturbation). In SOS network we can still see this trend. But in fission yeast network, all methods, except the random method, performs equally well before step 5. In general, we can conclude that sampling method is still very powerful when the number of initial condition is constrained.

## Discussion

In this paper, we discuss a reverse engineering approach to reconstruct biological regulatory network from experiments. Instead of using real data from experiments, we applied three well-known regulatory networks (budding yeast cell cycle, fission yeast cell cycle, and *E. coli.* SOS network) as tests to generate the trajectories of the networks. We focus on how to design the experiment (in this study, the initial conditions) to get the most useful information from data of evolution trajectory. For this purpose, we propose three methods, namely, maximum distance method, trajectory entropy method, and sampling method, to derive the optimal initial conditions for experiments. The performance of these methods is discussed and evaluated by comparing the reconstructed networks with the original ones in three known systems. We also test our methods under more realistic circumstance when the number of nodes that can be perturbed is limited. From these analyses we conclude that the sampling method is the best among the three methods. Two issues are called for further investigation.

First, the methods discussed in this paper are based on the assumption that all the undetermined networks have equal probability to be the true regulatory network. In fact, it is becoming more and more clear that biological regulatory networks have a strong bias toward a small set that shows dynamical robustness and structural coherence [15], [16], [17]. How to include this information in our calculation is a challenge. The researches in this area are underway in our laboratory.

Second, choosing different initial conditions to get different trajectories of underlying biological regulatory networks may not be practical in experiments, because certain initial conditions are not attainable. Though we have discussed the performance of our methods when states of limited number of nodes can be modulated, sometimes not all the combinations of the modulation are possible. Also in real microarray expression experiments, different data set is usually obtained by knocking out or knocking down one or few genes, rather than choosing different initial conditions. In this sense, the work reported here just provides a general method in principle. It shows the possibility of using the reverse engineering methods to optimize microarray expression experiments for the construction of biological regulatory networks. The actual methods must be modified and improved to meet the needs of experimentalists.
